# An Unsupervised Learning-Based Multi-Organ Registration Method for 3D Abdominal CT Images

**DOI:** 10.3390/s21186254

**Published:** 2021-09-18

**Authors:** Shaodi Yang, Yuqian Zhao, Miao Liao, Fan Zhang

**Affiliations:** 1 School of Automation, Central South University, Changsha 410083, China; ysdjane@csu.edu.cn (S.Y.); zhangfan219@csu.edu.cn (F.Z.); 2 Hunan Xiangjiang Artificial Intelligence Academy, Changsha 410083, China; 3 Hunan Engineering Research Center of High Strength Fastener Intelligent Manufacturing, Changde 415701, China; 4 School of Computer Science and Engineering, Hunan University of Science and Technology, Xiangtan 411201, China; mliao@hnust.edu.cn

**Keywords:** registration, convolutional neural network, medical image, abdominal CT

## Abstract

Medical image registration is an essential technique to achieve spatial consistency geometric positions of different medical images obtained from single- or multi-sensor, such as computed tomography (CT), magnetic resonance (MR), and ultrasound (US) images. In this paper, an improved unsupervised learning-based framework is proposed for multi-organ registration on 3D abdominal CT images. First, the explored coarse-to-fine recursive cascaded network (RCN) modules are embedded into a basic U-net framework to achieve more accurate multi-organ registration results from 3D abdominal CT images. Then, a topology-preserving loss is added in the total loss function to avoid a distortion of the predicted transformation field. Four public databases are selected to validate the registration performances of the proposed method. The experimental results show that the proposed method is superior to some existing traditional and deep learning-based methods and is promising to meet the real-time and high-precision clinical registration requirements of 3D abdominal CT images.

## 1. Introduction

Sensors and sensing systems play important roles in various medical applications, including disease diagnosis, monitoring, preoperative planning, surgical navigation, and so on [1,2,3,4]. Registration is one of the fundamental technologies that enable the sensing systems to be used in the above-mentioned applications [5,6,7,8]. Abdominal images taken from inter individuals are different in shape and texture, due to the complex intensity distribution of multi-organ, susceptibility of respiratory movement, and so on. Most existing methods cannot simultaneously meet the clinical requirements of high-accuracy and real-time performance for full abdominal image registration. To solve the above problems, many researchers pay attention to the segmented-based abdominal image registration methods. For example, Li et al. [9] proposed a liver MR image registration method based on the respiratory sliding motion segmentation that achieves more accurate registration results. Xie et al. [10] proposed a lung and liver 4D-CT image registration method based on tissue features and ROI segmentation, which can be implemented on clinical data.

In clinical practice, experts usually delineate the regions of interest (ROIs) of the target organ and organs at risk for treatment planning, and thereby the multi-organ ROIs of the abdominal medical images can be naturally obtained. For the registration stage, the traditional registration methods usually obtain the optimized deformation field by iteratively minimizing the custom energy function including data and regularization terms [11,12,13,14], and the deformation field of each pair of fixed and moving images is calculated independently by the traditional methods. The registration time of the traditional methods is always substantial, especially when the pair-wise images have a large anatomical difference. For example, the registration time of the methods including Demons [15], Elastix [16], and free-form deformation with b-splines [17] on medical images are ranged from minutes to hours.

To address this issue, many researchers have begun to pay more attention to the learning-based registration methods implemented by convolutional neural networks (CNNs), which can be divided into supervised and unsupervised [18,19,20,21]. These learning-based methods use the CNN model to obtain good initialization parameters for medical image registration. Most supervised methods require ground truth of deformation fields or anatomical segmentation masks, which are obtained from the traditional registration tools or manual delineation. These approaches entail much effort on data labeling, and the registration performance is influenced by the quality of the labels.

The unsupervised methods can directly use the unlabeled data to train CNNs model, avoiding the expensive and time-consuming labeling work. For example, Lei et al. [22] presented a multi-scale unsupervised registration framework for abdominal 4D-CT images. It has three loss functions to train the global and local subnetworks, including the similarity, adversarial, and regularization losses. Heinrich et al. [23] used a discrete displacement layer to improve the accuracy of the unsupervised learning-based 3D abdominal CT image registration framework. Balakrishnan et al. [24] proposed a U-net unsupervised registration framework, namely Voxelmorph, for 3D brain MR images. The regular similarity and regularization loss functions are used to train the framework, and then an auxiliary data loss function is added in the testing stage. Zhao et al. [25] proposed a U-net unsupervised registration framework for liver CT and brain MR images, namely VTN. Specifically, the affine transformation is integrated into the framework as a subnetwork to reduce the pre-processing time. Subsequently, the recursive cascaded networks (RCNs) by Zhao et al. [26] can be embedded into any base network as general architecture. Both Kuang et al. [27] and Mok et al. [28] emphasized that the distortion of the transformation field is non-negligible. They integrated the topology-preserving loss into the total loss functions for preventing the distortion.

Aiming at avoiding the time-consuming pre-processing and maintaining the topology-preserving property of the transformation, we developed an unsupervised learning-based registration framework for the segmented multi-organ from 3D abdominal CT images. First, the recursive cascaded network (RCN) modules are embedded into a basic U-net framework for promoting the unsupervised end-to-end learning. Secondly, the affine transformation subnetwork is cascaded with the subsequent fine registration subnetworks to implement the integration of the transformation field prediction from coarse to fine. Then, a topology-preserving loss is added in the total loss function for the training the registration framework, and then the transformation field is obtained for abdominal multi-organ registration.

The main contributions of this paper are as follows. First, an unsupervised learning-based registration framework is proposed, which can automatically learn from unlabeled data avoiding the time-consuming expert labeling work. Secondly, the coarse-to-fine RCN modules are embedded into the framework, which can efficiently deal with the large-scale deformation and improve the accuracy of the transformation field prediction. Moreover, an additional loss is integrated into the total loss function, which can ensure the registration with the property of the topology preservation. Finally, the proposed method proved to be more precise and faster than some existing registration methods on multi-organ from 3D abdominal CT images.

## 2. Methods

The essential of image registration is to find the mapping relationship between the fixed image $I_{f}$ and the moving image $I_{m}$, which enables the $I_{m}$ align to the $I_{f}$ with a reasonable transformation T. Generally, the energy function of optimizing T is as follows [29]:

(1) $$\hat{T}=\arg\underset{T}{\min}L_{S}(I_{f},I_{m}\circ T)+\lambda L_{R}(T)$$

where $L_{S}$ denotes the similarity term, $L_{R}$ represents the regularization term to constraint the $L_{S}$, and λ is the empirical constant. The uniform image domain of $I_{m}$, $I_{f}$, and T is Ω→ℝ in *D*-dimension, where the value of *D* is 3.

### 2.1. Optimization Problem Formulation

**Figure 1:**
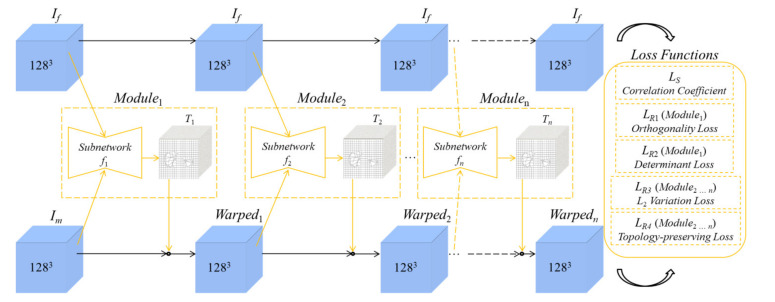
Framework of the proposed method.

In this paper, the optimal parameters for transformation T are estimated by an improved unsupervised learning-based model. The task of the model is to use a flow prediction function $F(I_{m},I_{f})$ to obtain the transformation field $T(I_{m},I_{f})$ during a recursive procedure. As shown in Figure 1, there are *n* modules cascaded in the model. Each module contains a predicted transformation field T in a subnetwork. Therefore, the final warped $I_{m}$ can be written as [25]:

(2) $$Warped_{m}^{\left(n\right)}=I_{m}\circ F(I_{m},I_{f})$$

and the output of the model is composited by:

(3) $$F(I_{m},I_{f})=T_{1}\circ \cdots \circ T_{n}$$

where $T_{1}$, …, $T_{n}$ represent the transformation field of the modules from 1st to *n*th, respectively. For instance, the kth predicted transformation field $T_{k}$ can be represented as $T_{k}=f_{k}(Warped_{m}^{\left(k-1\right)},I_{f})$, where $f_{k}$ is the kth prediction function of F. Therefore, the moving image is gradually warped by the modules.

### 2.2. Architecture of the Unsupervised Learning-Based Networks

First, we embed the coarse-to-fine modules of RCNs into the basic U-net framework, intending to improve its registration performance on multi-organ from 3D abdominal CT images. The RCN modules can be used to successively predict their corresponding flow field f, as shown in Figure 1. Then, we integrate a topology-preserving loss into the total loss function to avoid the distortion of the predicted transformation T of the registration framework.

#### 2.2.1. Coarse Registration

Affine transformation is widely applied to coarsely register the pair-wise medical images as pre-processing because it can reduce the registration errors caused by the difficulty of predicting the large deformation between the two images. Researchers commonly use the conventional software to perform the above process in a traditional way. However, it is time-consuming and requires researchers’ manual operations. To solve the above problems, the framework of the proposed method assigns the first subnetwork (namely coarse-subnetwork), to predict the affine transformation field with a small computational burden. The coarse-subnetwork contains a series of downsampling operations followed by a fully connected layer. The architecture of the network is the same as that of [25]. First, the input images are sequentially resampled to 64^3^, 32^3^, 16^3^, 8^3^, and 4^3^ by the convolution layers with the uniform kernel size as 3^3^ and stride as 2. Then, the flow transformation field is predicted according to the output parameters of the fully connected layer. The flow transformation field is represented as [25]:

(4) T(x)=Ax+b

where x denotes the voxel in the image domain Ω, A is a transform matrix, and b is a displacement vector. The moving image $I_{m}$ is first warped by the predicted affine transformation parameters, and then becomes the initial input for the fine registration subnetworks (namely fine-subnetworks).

#### 2.2.2. Fine Registration

For fine registration, each subnetwork has the same encode–decoder architecture as U-net [30]. Both of them contain five resolutions to obtain multiple receptive fields, including 64^3^, 32^3^, 16^3^, 8^3^, and 4^3^. The skip connection is used to concatenate the features of the same resolution from the encoding to the decoding stages, enabling the subnetwork to obtain more accurate predictions for the transformation field. For a pair of images, the transformation prediction is implemented by first extracting features in the encoding stage, and then restoring the image resolution in the decoding stage. The uniform kernel size and stride of the subnetworks are 3^3^ and 2, respectively. All the subnetworks are used to progressively predict the flow transformation field for fine registration except the coarse-subnetwork.

### 2.3. Loss Functions

The unsupervised learning-based networks are trained by minimizing the following loss function:

(5) $$L_{\mathrm{Total}}=L_{\mathrm{Coarse}}+L_{\mathrm{Fine}}$$

(6) $$L_{\mathrm{Coarse}}=L_{S}+\lambda_{1}L_{R1}+\lambda_{2}L_{R2}$$

(7) $$L_{\mathrm{Fine}}=L_{S}+\lambda_{3}L_{R3}+\lambda_{4}L_{R4}$$

where $L_{\mathrm{Total}}$ denotes the total loss, $L_{\mathrm{Coarse}}$ and $L_{\mathrm{Fine}}$ represent the subtotal losses for the coarse- and the fine-subnetworks, respectively. $L_{S}$ is the similarity loss of both $L_{\mathrm{Coarse}}$ and $L_{\mathrm{Fine}}$. $L_{R1}$ to $L_{R4}$ are the regularization terms of their corresponding subtotal loss functions. $\lambda_{1}$ to $\lambda_{4}$ are the empirical constants.

#### 2.3.1. Similarity Loss

For mono-modal medical images, the correlation coefficient (CC) is suitable to be the similarity loss $L_{S}$ both for $L_{\mathrm{Coarse}}$ and $L_{\mathrm{Fine}}$, which is defined as:

(8) $$L_{S}=1-CC(I_{f},I_{m}\circ T)$$

(9) $$CC[I_{f},I_{m}\circ T]=\frac{\sigma [I_{f},I_{m}\circ T]}{\sqrt{\sigma [I_{f},I_{f}]}\sqrt{\sigma [I_{m}\circ T,I_{m}\circ T]}}$$

where σ[•] denotes the covariance. The values of CC ranges from −1 to 1, indicating that the degree of correlation between two images changes from completely anti-correlated to correlated.

#### 2.3.2. Regularization Terms for the Coarse-Subnetwork

Dealing with the large-scale deformation, we first employ the coarse-subnetwork to implement the affine transformation of the moving image $I_{m}$. To avoid over non-rigid transformation field prediction, the orthogonality loss $L_{R1}$ and determinant loss $L_{R2}$ are used to regularize the similarity loss $L_{S}$ in the coarse-subnetwork, as shown in Equation (6). The $L_{R1}$ and $L_{R2}$ are respectively defined as [25,26]:

(10) $$L_{R1}=-6+\sum_{i=1}^{3}\mu_{i}^{2}+\mu_{i}^{-2}$$

(11) $$L_{R2}={\left(-1+\det(A+I)\right)}^{2}$$

where $\mu_{i}$ is the singular values obtained from A+I, $i=\left\{1,2,3\right\}$. I is the identity matrix, and A is the affine transform matrix.

#### 2.3.3. Regularization Terms for the Fine-Subnetworks

Generally, researchers only use a regular regularization term to penalize the similarity measure, such as *L*_1_ norm, *L*_2_ norm, and total variation. The smoothness property of the transformation field can be maintained by the above-mentioned term. First, we combine the similarity loss $L_{S}$ and the smooth loss $L_{R3}$ as a conventional group for transformation field prediction. However, one of the desirable properties of the transformation field is always ignored, namely topology-preserving. The relevant regularization term imposing on the similarity measure can prevent the distortion of the transformation field. Therefore, we integrate the topology-preserving loss $L_{R4}$ into the conventional group, aiming to obtain a more physically possible and accurate transformation field, as shown in Equation (7). The *L*_2_ variation loss $L_{R3}$ is defined as [31]:

(12) $$L_{R3}=\frac{1}{3N}\sum_{x}\sum_{i=1}^{3}{\left(T(x+\rho_{i})-T(x)\right)}^{2}$$

where N is the total number of voxel x, $\rho_{i}$ forms the basis of ℝ, $i=\left\{1,2,3\right\}$.

Since the Jacobian determinant or its variations are commonly used to evaluate the topology preservation of the dense vector field, they can be used as a regularization loss for avoiding the distortion of the transformation field. Therefore, we use the negative Jacobian determinants as the topology-preserving loss $L_{R4}$ to further constrain the similarity loss of the fine-subnetworks. The $L_{R4}$ is defined as [27,28]:

(13) $$L_{R4}=\frac{1}{N}\sum_{x\in \Omega }\sigma (\left|J_{T}(x)\right|-J_{T}(x))$$

where N is the total number of voxel x, $J_{T}(x)$ denotes the Jacobian determinant; σ(•) is defined as max(0,•) that can linearly activate the positive values and change the others from negative to zero. Therefore, σ(•) decides whether $L_{R4}$ will penalize x. If the orientation of x is inconsistent with those of the neighbors, $L_{R4}$ can be activated (and vice versa).

## 3. Results and Discussion

### 3.1. Database

We selected 30, 22, 19, and 5 abdominal CT volumes from the publicly available training datasets from BTCV [32], LiTS [33], Sliver07 [34], and 3Dircadb [35], respectively, to validate the proposed method. The original size of the volumes is 512×512×depth.

BTCV is a dataset for the performance comparisons of 3D abdominal CT image segmentation methods. Its training dataset contains 30 objects with the liver, left kidney, right kidney, and spleen segmentation masks. All of them were selected for this study.

LiTS is a challenging dataset for liver tumor segmentation. A total of 130 training objects are provided with the liver and tumor segmentation masks. In this study, our experts randomly selected 22 objects and manually supplemented the corresponding left kidney, right kidney, and spleen masks.

Sliver07 is a challenging dataset for liver segmentation. Its training dataset includes 20 objects with liver segmentation masks. First, one of the objects was excluded considering the unclear structure of the spleen. Then, the remaining 19 objects were selected for this study, and our experts manually supplemented their left kidney, right kidney, and spleen masks.

3Dircadb is a 3D abdominal CT images dataset. It contains 20 training objects with multiple structures’ segmentation masks, including liver, left kidney, liver tumor, and so on. In this study, six objects were selected that simultaneously include liver, left kidney, right kidney, and spleen segmentation masks.

Therefore, 76 abdominal multi-organ CT volumes were formed by combining the above selected original volumes with their masks. One of them was used as the atlas (i.e., the fixed image), which is randomly selected from LiTS. A total of 15 volumes were evenly selected from LiTS, BTCV, and Sliver07, and paired with the atlas as the testing groups. The remsining 60 volumes were paired with the atlas as the training groups. The training and testing data were unified to 1283 due to the limited memory usage of the GPU (NVIDIA RTX 2080 Ti, 11G).

### 3.2. Evaluation Indexes

For registration analysis, the global intensity differences between two images are first evaluated by the root mean squared error (RMSE) and the peak signal-to-noise ratio (PSNR) [36]:

(14) $$RMSE(I_{f},I_{m}\circ T)=\sqrt{MSE}$$

(15) $$PSNR=10\times \log_{10}(\frac{MAX_{I}^{2}}{MSE})$$

where N is the total number of voxel x. $MAX_{I}$ is the maximum possible voxel value of the image, $MSE=\frac{1}{N}{\sum_{x\in \Omega }\left(I_{f}(x)-I_{m}\circ T(x)\right)}^{2}$.

Secondly, the local intensity differences between two images are evaluated by the structural similarity index (SSIM) [36]:

(16) $$SSIM(I_{f},I_{m}\circ T)=\frac{\left(2\mu_{I_{f}}\mu_{I_{m}\circ T}+c_{1})(2\sigma_{I_{f}I_{m}\circ T}+c_{2}\right)}{\left(\mu_{I_{f}}^{2}+\mu_{I_{m}\circ T}^{2}+c_{1})(\sigma_{I_{f}}^{2}+\sigma_{I_{m}\circ T}^{2}+c_{2}\right)}$$

where $\mu_{I_{f}}$ and $\mu_{I_{m}\circ T}$ are the mean values, $\sigma_{I_{f}}^{2}$ and $\sigma_{I_{m}\circ T}^{2}$ are the variances, and $\sigma_{I_{f}I_{m}\circ T}$ is the covariance for $I_{f}$ and $I_{m}\circ T$; $c_{i}={\left(k_{i}L\right)}^{2}$ denotes the constant, which $i=\left\{1,2\right\}$, $k_{1}=0.01$, $k_{2}=0.03$, and L is the range of voxel value.

Moreover, the dice coefficient (DICE), Haustorff distance (HD), contour mean distance (CMD), intersection over union (IOU), sensitivity (SS), and specificity (SC) are also included [37]:

(17) $$DICE=\frac{X\cap Y}{\left|X\right|+\left|Y\right|}$$

(18) $$HD=\max\left\{h(X,Y),h(Y,X)\right\}$$

(19) $$CMD=\max\left\{h(X_{b},Y_{b}),h(Y_{b},X_{b})\right\}$$

(20) $$IOU=\frac{TP}{TP+FN+FP}$$

(21) $$SS=\frac{TP}{TP+FN}$$

(22) $$SC=\frac{TP}{TN+FP}$$

where X and Y are the multi-organ segmentation masks of the fixed and warped images, respectively; $X_{b}$ and $Y_{b}$ are the boundaries for X and Y, respectively TP, TN, FN, and FP is the true positive, true negative, false negative, and false positive voxels in the multi-organ segmentation masks of the fixed and warped images, respectively. $h(X,Y)=\underset{x\in X}{\max}\underset{y\in Y}{\min}\left\|x-y\right\|$ is the distance from X to Y, and $h(X_{b},Y_{b})=\underset{x\in X_{b}}{\max}\underset{y\in Y_{b}}{\min}\left\|x-y\right\|$ the distance from $X_{b}$ to $Y_{b}$.

### 3.3. Experimental Analysis

#### 3.3.1. Internal Comparisons

We use the basic U-net (namely Base-Net) integrated with our total loss function to explore the optimal number of the RCN modules. First, the coarse-subnetwork-related module is integrated into the Base-Net without counting. Then, the Base-Net is cascaded with 1, 2, 3, 5, and 7 RCN modules, namely Base-Net-1, Base-Net-3, Base-Net-5, and Base-Net-7, respectively, for experimental analysis. The maximum number of the RCN modules is determined by the memory usage of the GPU. Subsequently, we chose the best performing model as the proposed method for external comparisons, i.e., Base-Net-7. The values of $\lambda_{1}$ and $\lambda_{2}$ are the same as those in [25], and the value of $\lambda_{4}$ is the same as that in [27], i.e., $\lambda_{1}=10^{-1}$, $\lambda_{2}=10^{-1}$, and $\lambda_{4}=10^{-5}$. The optimal value of $\lambda_{3}$ is selected according to the experimental results. The uniform batch size, epoch, and learning rate of these comparison models are set as 2, 5, and 10^−^^4^, respectively.

**Figure 2:**
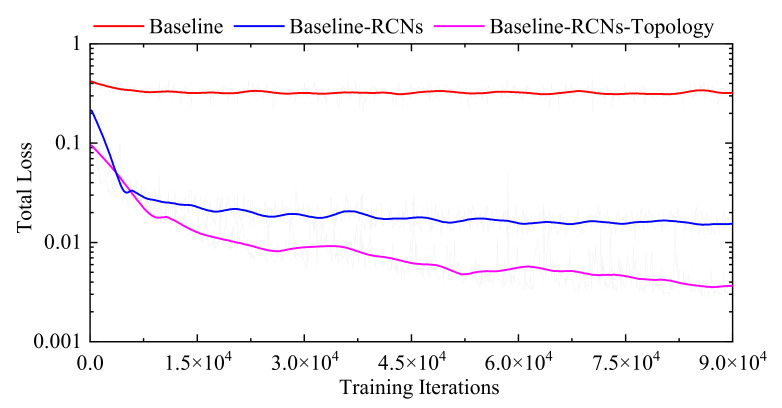
Total loss curves of different models.

**Figure 3:**
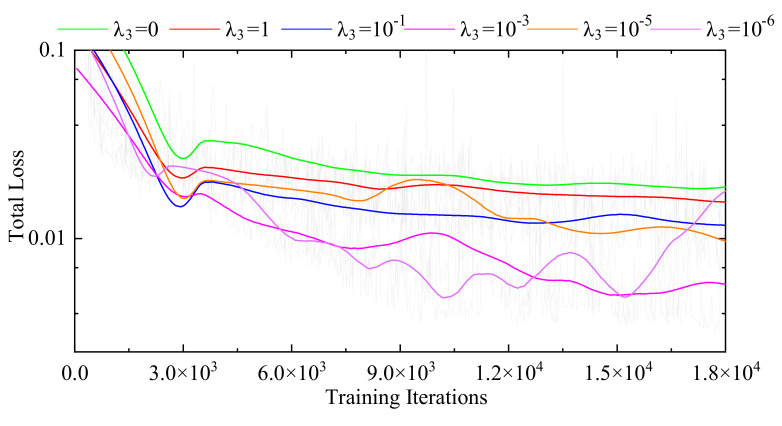
Total loss curves of the Base-Net-7 with different values of $\lambda_{3}$.

Figure 2 shows the total loss trends of different models on the training dataset. The curves of the Baseline and Baseline-RCNs-Topology are obtained from the Base-Net and the Base-Net-7, respectively. The curve of the Baseline-RCNs is acquired from the same model as the Base-Net-7 without the topology-preserving loss. Compared with the Baseline, the Baseline-RCNs has lower total loss values, indicating that the RCN modules are effective in decreasing the registration errors; meanwhile, the Baseline-RCNs-Topology proves that the topology-preserving loss function modules can further improve the multi-organ registration performance on 3D abdominal CT images. Furthermore, Figure 3 displays the total loss trends of the Base-Net-7 with different values of $\lambda_{3}$. It can be seen that the Base-Net-7 with $\lambda_{3}=10^{-5}$ achieves the optimal performance on the training dataset. Therefore, the value of $\lambda_{3}$ is set as $10^{-5}$ in the proposed method.

Table 1 presents the internal comparisons of embedding different number of the RCNs modules into our Base-Net. As observed, the average values of the RMSE, HD, and CMD obtained by the Base-Net-1, 3, and 7 are smaller than those of the Base-Net. Meanwhile, the values of the PSNR, SSIM, DICE, IOU, SS, and SC obtained by the Base-Net-1, 3, 5, and 7 are higher than those of the Base-Net. It may be because the architecture of the Base-Net is changed to a recursive one by embedding the RCN modules, and the improved Base-Net can progressively predict a more accurate transformation field. Besides, the RMSE, HD, and CMD from Base-Net-7 are 54.01%, 31.45%, and 82.01% lower than those of the Base-net, respectively. The PSNR, SSIM, DICE, IOU, SS, and SC from Base-Net-7 are 38.16%, 7.92%, 33.81%, 65.66%, 17.52%, and 48.29% higher than those of the Base-Net, respectively. Moreover, the Base-Net-7 outperforms all of the other models on the multi-organ registration from 3D abdominal CT images. It can be therefore concluded that the optimal number is seven for embedding the RCNs modules into the Base-Net.

**Figure 4:**
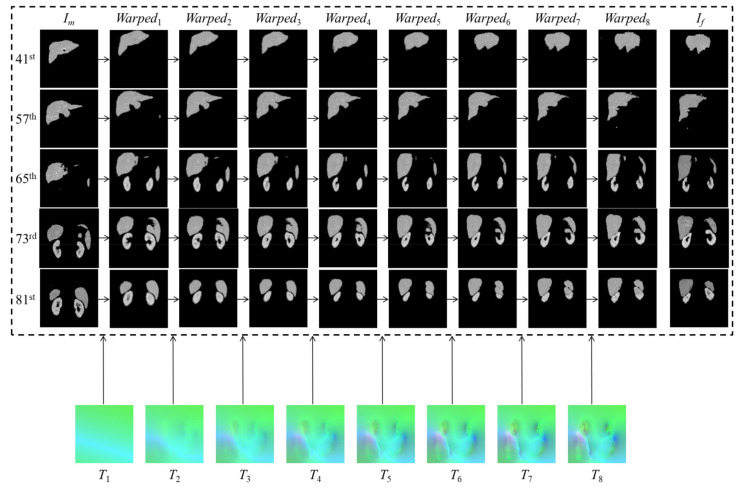
Visualization of a randomly selected testing group on the proposed method.

Figure 4 displays the registration progress of the proposed method on the 41st, 57th, 65th, 73rd, and 81st slices of the randomly selected testing group, where $T_{1}$ is the affine transformation field, and $T_{2}$ to $T_{8}$ are the transformation field obtained by 1st to 7th RCN modules, respectively. It can be observed that there exist big intensity differences between the fixed image $I_{f}$ and the moving image $I_{m}$. However, the RCN modules can help the model progressively obtain the final warped images, which are exactly similar to the fixed images. Therefore, the proposed method has good performance on multi-organ registrations from 3D abdominal CT images.

#### 3.3.2. External Comparisons

We compared our method against three traditional methods, Demons [38], Hybrid [39], MSI [40], and two state-of-art unsupervised learning-based methods, VTN [25] and Voxelmorph [24]. We ran the experimental methods on an Intel i5-GTX1060 CPU and an NVIDIA RTX 2080 Ti GPU, respectively.

Table 2 shows the external comparisons of the abdominal multi-organ registration results on different methods. As observed, the proposed method performs comparably to MSI in terms of RMSE, and is superior to Demons, Hybrid, VTN, and Voxelmorph. Moreover, the average values of PSNR, SSIM, DICE, IOU, SS, SC, HD, and CMD from the proposed method are 29.5584, 0.9732, 0.9775, 0.9562, 0.9982, 0.9578, 12.2646, and 3.9296, respectively, which are better than those of the traditional and unsupervised learning-based methods. The registration time in Table 2 illustrates that the unsupervised learning-based methods are obviously faster than the traditional ones. Although VTN achieves the shortest registration time, its performances on the other evaluation indicators are barely satisfactory. Generally, the proposed method gives a good compromise to meet the real-time and high-accuracy clinical requirements.

**Figure 5:**
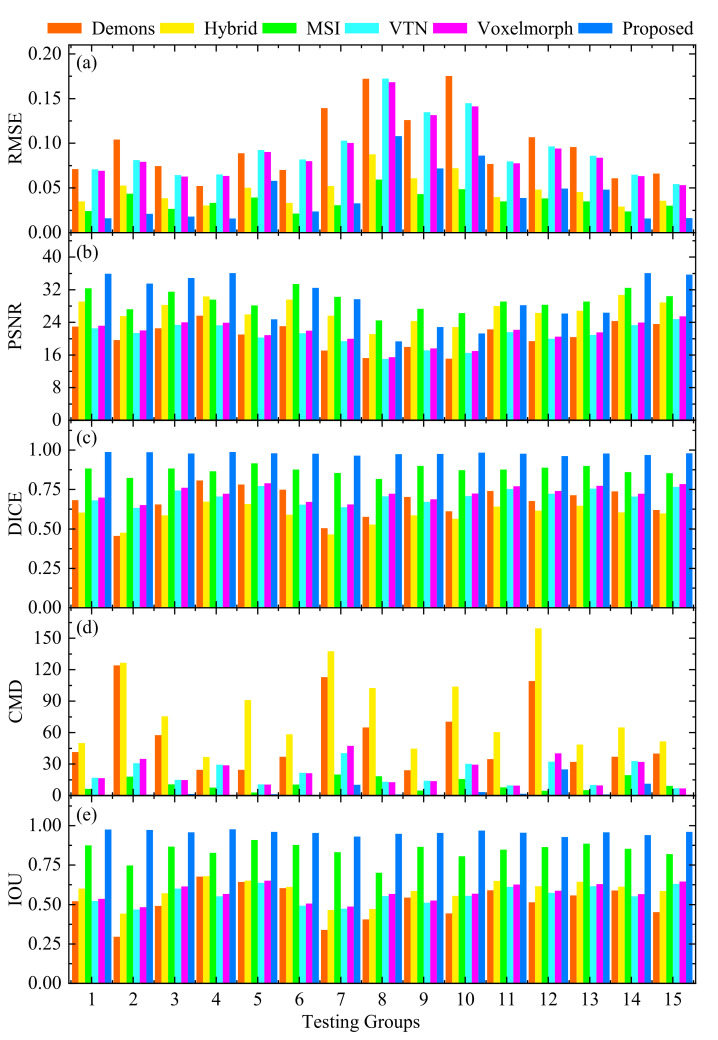
Evaluation metrics of 15 pair-wise testing groups’ registration results with different methods. (**a**) RMSE; (**b**) PSNR; (**c**) DICE; (**d**) CMD; (**e**) IOU.

**Figure 6:**
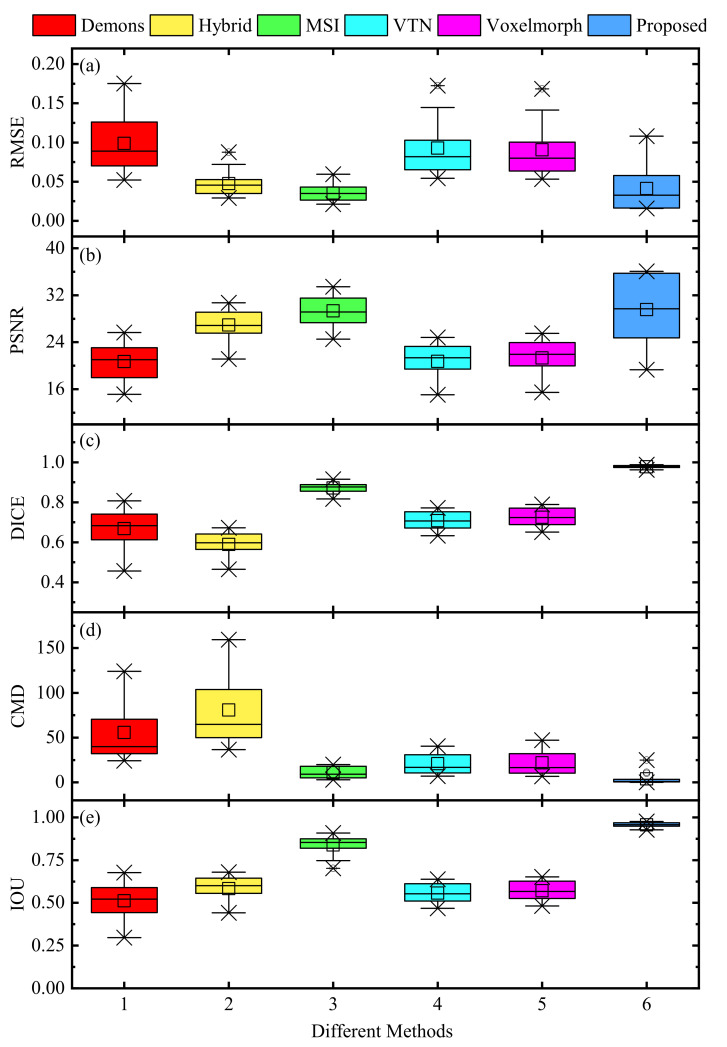
Boxplots for evaluation metrics of 15 pair-wise testing groups’ registration results with different methods. (**a**) RMSE; (**b**) PSNR; (**c**) DICE; (**d**) CMD; (**e**) IOU.

Figure 5 and Figure 6 directly display the histograms and boxplots of the evaluation metrics of 15 pair-wise testing groups’ registration results with different methods, respectively. It can be found that the distributions of the RMSE, PSNR, DICE, CMD, and IOU values in Figure 5 and Figure 6 are consistent with those in Table 2. Hence, the proposed method has stable registration performance, and is superior to the other competing methods in the above evaluation indicators.

**Figure 7:**
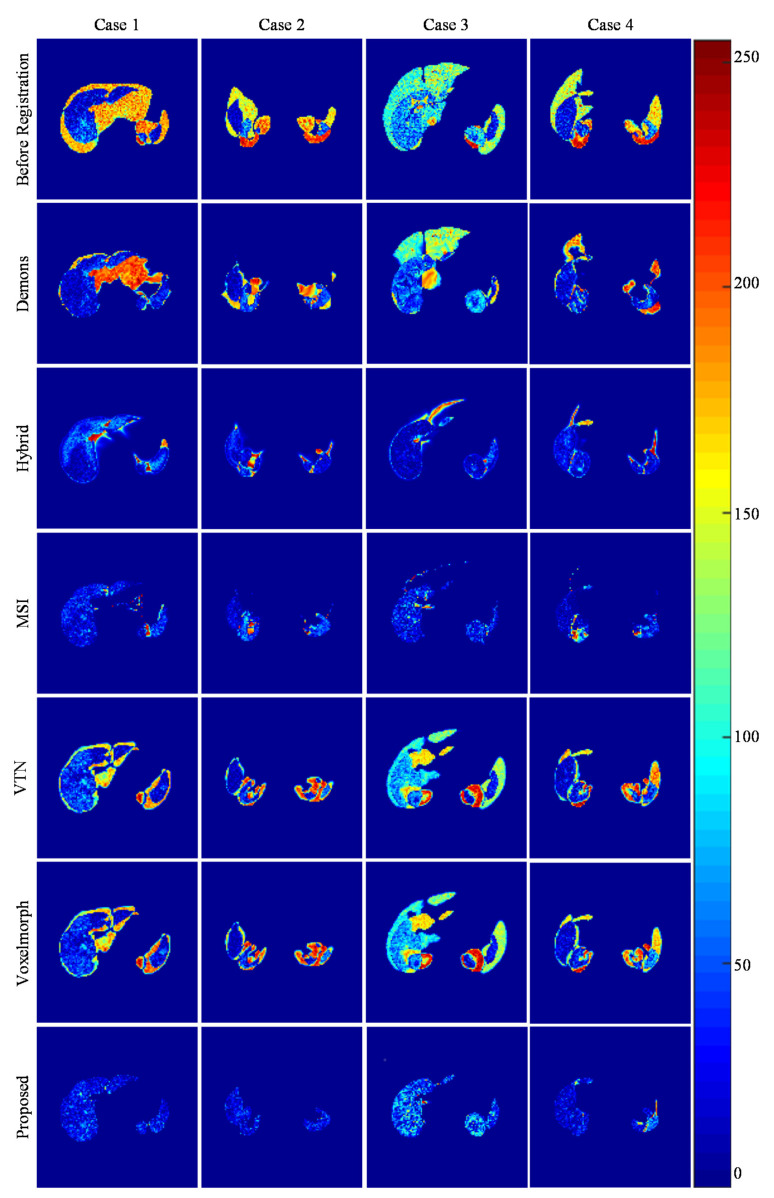
The intensity differences between the fixed and moving images from the paired testing groups with different methods.

Figure 7 presents the intensity differences between the fixed and moving images from four randomly selected testing groups. The intensity values of the image range from 0 to 255 and its corresponding color varies from blue to red. As shown in Figure 7, all pair-wise groups have significant differences before registration. Then, the Demons, VTN, and Voxelmorph methods produce considerable differences, indicating that their performances may be influenced by the noise and artifacts in the abdominal CT images. Moreover, the proposed method produces the smallest differences and outperforms the other competing methods. It can be concluded that our method can avoid the impact of the noise and artifacts and perform more accurately and robustly on multi-organ registration for 3D abdominal CT images.

## 4. Conclusions

In this paper, we present an improved unsupervised learning-based framework for multi-organ registration from 3D abdominal CT images. The coarse-to-fine RCNs modules are embedded into a basic U-net model, which can hence inherit the advantages of the recursive model and achieve better performance on multi-organ registration from 3D abdominal CT images. In addition, a topology-preserving loss is added in the total loss function, which can penalize the similarity loss to avoid the distortion of the predicted transformation field. The experimental results show that the proposed method has the optimal PSNR, SSIM, DICE, IOU, SS, SC, HD, and CMD average values and is competitive with the traditional and unsupervised learning-based methods, and therefore has the potential to be used in clinical practice.

## Figures and Tables

**Table 1:** The average metrics of abdominal multi-organ registration results with the different number of RCN modules.

Metric	Base-Net	Base-Net-1	Base-Net-3	Base-Net-5	Base-Net-7
RMSE	0.0898	0.0516	0.0430	0.0419	0.0413
PSNR	21.3938	26.8639	28.9574	29.3259	29.5584
SSIM	0.9018	0.9572	0.9701	0.9720	0.9732
DICE	0.7305	0.9155	0.9621	0.9724	0.9775
IOU	0.5772	0.8451	0.9272	0.9445	0.9562
SS	0.8494	0.9757	0.9944	0.9974	0.9982
SC	0.6459	0.8631	0.9320	0.9468	0.9578
HD (mm)	17.8924	15.0090	13.1231	12.6245	12.2646
CMD (mm)	21.8383	7.0733	4.7972	4.3341	3.9296

**Table 2:** The average metrics of abdominal multi-organ registration results with different methods.

Metrics	Demons	Hybrid	MSI	VTN	Voxelmorph	Proposed
RMSE	0.0987	0.0474	0.0355	0.0928	0.0906	0.0413
PSNR	20.6969	26.9121	29.3418	20.7456	21.3212	29.5584
SSIM	0.8925	0.9412	0.9592	0.8769	0.9009	0.9732
DICE	0.6678	0.5891	0.8715	0.7078	0.7253	0.9775
IOU	0.5113	0.5833	0.8386	0.5569	0.5709	0.9562
SS	0.8077	0.9189	0.9268	0.8256	0.8450	0.9982
SC	0.5836	0.6137	0.8997	0.6268	0.6407	0.9578
HD (mm)	27.7843	30.7378	25.7123	18.1536	17.7319	12.2646
CMD (mm)	55.6456	80.7641	10.7022	20.8790	21.8432	3.9296
CPU (s)	381.0294	510.1189	-	7.5612	8.6247	9.8922
GPU (s)	-	-	536.4378	1.3998	1.5212	1.6371

## Data Availability

Not applicable.

## References

[1] Bielecki Z., Stacewicz T., Wojtas J., Mikołajczyk J., Szabra D., Prokopiuk A. (2018). Selected optoelectronic sensors in medical applications. Opto-Electron. Rev..

[2] David D.D.S., Parthiban R., Jayakumar D., Usharani S., RaghuRaman D., Saravanan D., Palani U. (2021). Medical Wireless Sensor Network Coverage and Clinical Application of Mri Liver Disease Diagnosis. Eur. J. Mol. Clin. Med..

[3] Gao L., Zhang G., Yu B., Qiao Z., Wang J. (2020). Wearable human motion posture capture and medical health monitoring based on wireless sensor networks. Measurement.

[4] Luo X., He X., Shi C., Zeng H.-Q., Ewurum H.C., Wan Y., Guo Y., Pagnha S., Zhang X.-B., Du Y.-P. (2019). Evolutionarily Optimized Electromagnetic Sensor Measurements for Robust Surgical Navigation. IEEE Sens. J..

[5] Kok E.N.D., Eppenga R., Kuhlmann K.F.D., Groen H.C., Van Veen R., Van Dieren J.M., De Wijkerslooth T.R., Van Leerdam M., Lambregts D.M.J., Heerink W.J. (2020). Accurate surgical navigation with real-time tumor tracking in cancer surgery. NPJ Precis. Oncol..

[6] Ahn S.J., Lee J.M., Lee D.H., Lee S.M., Yoon J.-H., Kim Y.J., Yu S.J., Han J.K. (2017). Real-time US-CT/MR fusion imaging for percutaneous radiofrequency ablation of hepatocellular carcinoma. J. Hepatol..

[7] Li K., Su Z., Xu E., Huang Q., Zeng Q., Zheng R. (2017). Evaluation of the ablation margin of hepatocellular carcinoma using CEUS-CT/MR image fusion in a phantom model and in patients. BMC Cancer.

[8] Radu C., Fisher P., Mitrea D., Birlescu I., Marita T., Vancea F., Florian V., Tefas C., Badea R., Ștefănescu H. (2020). Integration of Real-Time Image Fusion in the Robotic-Assisted Treatment of Hepatocellular Carcinoma. Biology.

[9] Li D., Zhong W., Deh K.M., Nguyen T.D., Prince M.R., Wang Y., Spincemaille P. (2018). Discontinuity Preserving Liver MR Registration with Three-Dimensional Active Contour Motion Segmentation. IEEE Trans. Biomed. Eng..

[10] Xie Y., Chao M., Xing L. (2009). Tissue Feature-Based and Segmented Deformable Image Registration for Improved Modeling of Shear Movement of Lungs. Int. J. Radiat. Oncol. Biol. Phys..

[11] Fu Y., Lei Y., Wang T., Curran W.J., Liu T., Yang X. (2020). Deep learning in medical image registration: A review. Phys. Med. Biol..

[12] Litjens G., Kooi T., Bejnordi B.E., Setio A.A.A., Ciompi F., Ghafoorian M., van der Laak J.A., van Ginneken B., Sánchez C.I. (2017). A survey on deep learning in medical image analysis. Med. Image Anal..

[13] Nazib A., Fookes C., Perrin D. (2018). A comparative analysis of registration tools: Traditional vs deep learning approach on high resolution tissue cleared data. arXiv.

[14] Villena-Martinez V., Oprea S., Saval-Calvo M., Azorin-Lopez J., Fuster-Guillo A., Fisher R.B. (2020). When Deep Learning Meets Data Alignment: A Review on Deep Registration Networks (DRNs). Appl. Sci..

[15] Thirion J.P. (1998). Image matching as diffusion process: An analogy with Maxwell’s demons. Med. Image Anal..

[16] Klein S., Staring M., Murphy K., Viergever M.A., Pluim J.P.W. (2009). elastix: A Toolbox for Intensity-Based Medical Image Registration. IEEE Trans. Med. Imaging.

[17] Modat M., Ridgway G., Taylor Z., Lehmann M., Barnes J., Hawkes D.J., Fox N., Ourselin S. (2010). Fast free-form deformation using graphics processing units. Comput. Methods Programs Biomed..

[18] Cao X., Yang J., Zhang J., Nie D., Kim M.-J., Wang Q., Shen D. (2017). Deformable image registration based on similarity-steered CNN regression. Proceedings of the International Conference on Medical Image Computing and Computer-Assisted Intervention.

[19] Ferrante E., Oktay O., Glocker B., Milone D.H. (2018). On the adaptability of unsupervised CNN-based deformable image registration to unseen image domains. Proceedings of the International Workshop on Machine Learning in Medical Imaging.

[20] Blendowski M., Hansen L., Heinrich M.P. (2021). Weakly-supervised learning of multi-modal features for regularised iterative descent in 3D image registration. Med. Image Anal..

[21] Xu Z., Niethammer M. DeepAtlas: Joint semi-supervised learning of image registration and segmentation. Proceedings of the International Conference on Medical Image Computing and Computer-Assisted Intervention.

[22] Lei Y., Fu Y., Wang T., Liu Y., Patel P., Curran W.J., Liu T., Yang X. (2020). 4D-CT deformable image registration using multiscale unsupervised deep learning. Phys. Med. Biol..

[23] Heinrich M.P., Hansen L. (2020). Highly Accurate and Memory Efficient Unsupervised Learning-Based Discrete CT Registration Using 2.5D Displacement Search. Proceedings of the International Conference on Medical Image Computing and Computer-Assisted Intervention.

[24] Balakrishnan G., Zhao A., Sabuncu M.R., Guttag J., Dalca A.V. (2019). VoxelMorph: A Learning Framework for Deformable Medical Image Registration. IEEE Trans. Med. Imaging.

[25] Zhao S., Lau T., Luo J., Chang E.I.-C., Xu Y. (2020). Unsupervised 3D End-to-End Medical Image Registration with Volume Tweening Network. IEEE J. Biomed. Health Inform..

[26] Zhao S., Dong Y., Chang E., Xu Y. Recursive cascaded networks for unsupervised medical image registration. Proceedings of the IEEE/CVF International Conference on Computer Vision.

[27] Kuang D., Schmah T. (2019). Faim—A convnet method for unsupervised 3d medical image registration. Proceedings of the International Workshop on Machine Learning in Medical Imaging.

[28] Mok T.C.W., Chung A. Fast symmetric diffeomorphic image registration with convolutional neural networks. Proceedings of the IEEE/CVF Conference on Computer Vision and Pattern Recognition.

[29] Ferrante E., Paragios N. (2017). Slice-to-volume medical image registration: A survey. Med. Image Anal..

[30] Ronneberger O., Fischer P., Brox T. (2015). U-net: Convolutional networks for biomedical image segmentation. Proceedings of the International Conference on Medical Image Computing and Computer-Assisted Intervention.

[31] Rudin L.I., Osher S., Fatemi E. (1992). Nonlinear total variation based noise removal algorithms. Phys. D Nonlinear Phenom..

[32] Xu Z., Lee C.P., Heinrich M.P., Modat M., Rueckert D., Ourselin S., Abramson R.G., Landman B.A. (2016). Evaluation of Six Registration Methods for the Human Abdomen on Clinically Acquired CT. IEEE Trans. Biomed. Eng..

[33] Bilic P., Christ P.F., Vorontsov E., Chlebus G., Chen H., Dou Q., Fu C.-W., Han X., Heng P.-A., Hesser J. (2019). The Liver Tumor Segmentation Benchmark (LiTS). arXiv.

[34] Heimann T., Ginneken B.V., Styner M.A. Segmentation of the Liver 2007(SLIVER07). http://sliver07.isi.uu.nl/.

[35] Soler L., Hosttettle A., Charnoz A., Fasquel J., Moreau J. 3D Image Reconstruction for Comparison of Algorithm Database: A Patient Specific Anatomical and Medical Image Database. https://www.ircad.fr/research/3dircadb/.

[36] Sara U., Akter M., Uddin M.S. (2019). Image quality assessment through FSIM, SSIM, MSE and PSNR—A comparative study. J. Comput. Commun..

[37] Pei H.-Y., Yang D., Liu G.-R., Lu T. (2021). MPS-Net: Multi-Point Supervised Network for CT Image Segmentation of COVID-19. IEEE Access.

[38] Lombaert H., Grady L., Pennec X., Ayache N., Cheriet F. (2014). Spectral Log-Demons: Diffeomorphic Image Registration with Very Large Deformations. Int. J. Comput. Vis..

[39] Chan C.L., Anitescu C., Zhang Y., Rabczuk T. (2017). Two and Three Dimensional Image Registration Based on B-Spline Composition and Level Sets. Commun. Comput. Phys..

[40] Aganj I., Iglesias J.E., Reuter M., Sabuncu M.R., Fischl B. (2017). Mid-space-independent deformable image registration. NeuroImage.

